# Recent Advances in the Knowledge of the Mechanisms of Leptin Physiology and Actions in Neurological and Metabolic Pathologies

**DOI:** 10.3390/ijms24021422

**Published:** 2023-01-11

**Authors:** María E. Casado, Roberto Collado-Pérez, Laura M. Frago, Vicente Barrios

**Affiliations:** 1 Department of Endocrinology, Hospital Infantil Universitario Niño Jesús, Instituto de Investigación La Princesa, E-28009 Madrid, Spain; 2 Centro de Investigación Biomédica en Red de Fisiopatología de la Obesidad y Nutrición (CIBEROBN), Instituto de Salud Carlos III, E-28029 Madrid, Spain; 3 Department of Pediatrics, Faculty of Medicine, Universidad Autónoma de Madrid, E-28029 Madrid, Spain

**Keywords:** adipokine, biomarkers, biochemical mechanisms, cancer, inflammation, leptin, metabolic regulation, microRNA, molecular biology, obesity

## Abstract

Excess body weight is frequently associated with low-grade inflammation. Evidence indicates a relationship between obesity and cancer, as well as with other diseases, such as diabetes and non-alcoholic fatty liver disease, in which inflammation and the actions of various adipokines play a role in the pathological mechanisms involved in these disorders. Leptin is mainly produced by adipose tissue in proportion to fat stores, but it is also synthesized in other organs, where leptin receptors are expressed. This hormone performs numerous actions in the brain, mainly related to the control of energy homeostasis. It is also involved in neurogenesis and neuroprotection, and central leptin resistance is related to some neurological disorders, e.g., Parkinson’s and Alzheimer’s diseases. In peripheral tissues, leptin is implicated in the regulation of metabolism, as well as of bone density and muscle mass. All these actions can be affected by changes in leptin levels and the mechanisms associated with resistance to this hormone. This review will present recent advances in the molecular mechanisms of leptin action and their underlying roles in pathological situations, which may be of interest for revealing new approaches for the treatment of diseases where the actions of this adipokine might be compromised.

## 1. Introduction

Leptin, a polypeptide hormone of 167 amino acids with an N-terminal secretory-signal sequence of 21 amino acids encoded by the *ob* gene, was originally described as a protector against obesity, since *ob*/*ob* mice (leptin-deficient) were obese [1]. This cytokine is mainly synthesized by adipocytes from white adipose tissue in proportion to fat stores, and, since its discovery by Friedman’s group in 1994, has been essentially studied in relationship to its crucial role as a regulator of energy balance via its actions on hypothalamic nuclei [2]. Leptin modulates energy homeostasis through its potent inhibitory effect on hypothalamic orexigenic neuropeptide Y/agouti-related peptide (NPY/AgRP)-coexpressing neurons and the activation of pro-opiomelanocortin (POMC)-expressing neurons in the arcuate nucleus [3]. NPY is necessary for some acute actions of AgRP on feeding behavior, mediated through its inhibitory effect on the melanocortin 4 receptor (MC4R) [4], whereas POMC and cocaine and amphetamine-related transcript (CART) neurons trigger MC4R neurons, especially those located in the paraventricular nucleus [5]. These cells play a key role in the control of food intake and energy balance, and the disruption of this circuit by changes in leptin sensitivity or mutations in the genes encoding leptin, the leptin receptor, POMC, or MC4R, amongst other genes in this system, can lead to severe obesity [6].

After the primary recognition for its role in modulating food intake and energy balance, this adipokine has emerged as a crucial molecule with pleiotropic functions. Much evidence indicates its implication in diverse physiological processes, such as glucose metabolism, hematopoiesis, bone remodeling, neurogenesis and neuroprotection, interaction with the immune system, reproduction, angiogenesis, blood pressure, gastric emptying, and glomerular filtration rate, among other functions [7]. Leptin is not only linked to physiological processes, but it is also involved in pathological situations. It is suggested to have anti-apoptotic and pro-angiogenetic effects [8] and to promote proliferation, cell survival and migration in physiological conditions, processes that may also affect the initiation and progression of different diseases, especially cancers of the brain, breast, or lung [9].

The involvement of leptin in pathological situations is more evident in disorders that involve an increase in serum leptin levels. In this sense, data shows a close relationship between obesity and different diseases, such as cancer or cardiac hypertrophy [10,11]. Liver and renal diseases are also associated with hyperleptinemia, both in humans and experimental models of obesity [12,13]. Decreased serum levels may be also related to lipodystrophy and associated pathologies, such as metabolic syndrome and liver diseases. In addition, hypoleptinemia seems to also be associated with adaptive responses, thus the reductions in this adipokine may be a starvation signal inducing hunger, which may be more potent than its effects as a physiological satiety factor [14].

Multiple signaling pathways activated after the binding of leptin to leptin receptors (ObRs) and inter-relations with other factors that may regulate levels of the receptor and vice versa could explain the plethora of leptin actions. Leptin is a class I adipocytokine that binds to at least five OBRs isoforms, all of which share the extracellular domain; however, the ObRb isoform has a well-conserved intracellular membrane-proximal proline-rich region that is essential for Janus kinase (JAK) 2 association, given that this receptor has no intrinsic kinase activity. Activation of the ObRb starts a cascade of signal transduction pathways, with the JAK/signal transducers and activators of transcription (JAK/STAT) pathway being the best studied. Leptin also increases JAK2-dependent activation of the extracellular signal-regulated kinase 1/2 (ERK1/2) mitogen-activated protein kinase (MAPK) [15] and the insulin receptor (IR) substrate (IRS)-phosphoinositide 3-kinase (PI3K)/Akt pathway. In addition, leptin exerts a role in the adenosine monophosphate-activated protein kinase (AMPK) and the cAMP-response element binding protein (CREB)-regulated transcription coactivator (CRTC) pathway [16].

Crosstalk of leptin with other hormones changes the signaling mentioned above, and stimulates the release of several cytokines, thus modifying the inflammatory state and influencing metabolic processes [17]. The synthesis and secretion of leptin are regulated by multiple factors, including various hormones such as insulin, steroid hormones, cytokines, norepinephrine, and glucocorticoids [18]. Our increased understanding of the mechanisms implicated in the control of its synthesis, its interaction with other cytokines, and signaling pathways has resulted in its recognition as an important etiological factor of metabolic, obesity, and other disorders, affecting not only the adipose tissue, but also other organs, including the brain, liver, and muscles, among others. Likewise, the reasonable manipulation of leptin or the use of leptin-sensitizing agents could be a promising possibility of therapy for obesity and related metabolic dysfunctions in the future.

## 2. Regulation of Leptin Efficiency

The actions of leptin are modulated at different levels. In this section, we will list the factors that regulate its levels, through transcription and biosynthesis, as well as its secretion. Subsequently, a summary of the hormones interacting with leptin signaling transduction, and in many cases linked to the regulation of the mRNA and protein levels of this adipokine will be specified. In addition, there is evidence that central leptin sensitivity is associated to its transport across the blood–brain barrier, indicating that the ability for leptin transport into the brain is reduced and this could be a significant cause for the in-adequate action of this hormone.

### 2.1. Leptin Signaling

Structural studies reveal that leptin is a member of the growth hormone four-helical cytokine subfamily, although primary sequence similarity between leptin and other long-chain helical cytokines do not appear to existent. Nevertheless, there is an unusual structural similarity. Leptin is a lengthened molecule with four antiparallel α-helices connected by two long crossover links. Several exposed hydrophobic residues in this adipokine appear to serve an important role in receptor binding. Two cysteine residues forming a di-sulfide bridge are essential for the stability and activity [19]. The amino acid sequence 39–42 in the first two loops is fundamental for ObR activation.

Three binding sites important for the interaction with its receptor have been characterized in the leptin molecule. The function of the first site is related to the formation of the complex and the subsequent initial activation of the ObR [20]. The second allows high-affinity binding between the ligand and the cytokine receptor homology (CRH) domain II, while the third binding site interacts with the receptor’s immunoglobulin (Ig)-like domain. The studies of Peelman et al. [21] suggest that leptin and its receptor could form an active hexameric complex formed by four receptor molecules and two leptin molecules, being the formation of the complex mediated through the first binding site.

Leptin exerts its actions in different organs that express the leptin receptor, such as the brain, adipose tissue, liver, muscle, or kidney, among others. The ObR is a single membrane spanning receptor of the class 1 cytokine receptor family and comprises six isoforms, produced by alternative splicing or ectodomain shedding, named ObRa to ObRf. Among these isoforms, isoform-b is the best characterized; it is the long-form with an intracellular domain of 302 amino acids and contains three highly conserved tyrosine residues (Y985, Y1077, Y1138) required for efficient leptin signaling. This long-form subtype mainly mediates the activation of critical second messenger pathways and normal leptin actions [22]. All of the isoforms share the same complex extracellular domain, consisting of two CRH domains separated by an Ig-like domain and followed by two membrane proximal fibronectin type III (FN III) domains. The CRH2 domain near the membrane is essential for leptin binding with high affinity, while the FN III domains have no affinity for leptin but are essential for activation of the receptor since mutation of two residues of cysteine in these domains block the signaling of this adipokine [22]. The Ig-like domain is also crucial for receptor activation since receptors that lack it can bind leptin but are unable to activate JAKs and the subsequent leptin signaling cascade. Mutations in the ObR cause obesity and hyperphagia in mice as well as hyperglycemia, insulin resistance, and fatty liver [23]. Furthermore, mutations in the human ObR produce obesity and pituitary dysfunction [24]. There are four short forms (ObRa, ObRc, ObRd, and ObRf), with cytoplasmic tails of 30–40 amino acids and one soluble form (ObRe) that acts as a leptin binding protein in plasma, playing an active role in the maintenance of body weight [25].

Leptin binding and dimerization leads to JAK2 tyrosine kinase phosphorylation of three tyrosine residues that serve as docking sites for the proteins Src homology 2 (SH2)-containing protein tyrosine phosphatase (SHP)2, signal transducer and activator of transcription (STAT)5, and STAT3. All JAKs have tandem kinase domains, which encode a pseudokinase (PK) and a tyrosine kinase (TK). Mapping of JAK mutants seems to indicate a two-step allosteric activation for cytokine receptors, where JAK molecules bound to monomeric cytokine receptors suffer a conformational equilibrium between an auto-inhibited state and an open state in which the PK is accessible for JAK dimerization. In this situation, the carboxyterminal tyrosine kinase domain is available to serve as an enzyme or substrate in a transphosphorylation event. In the absence of cytokine, equilibrium favors the closed state, establishing a low basal kinase activity. For cytokine-mediated receptor dimerization, the equilibrium is moved to the PK-mediated dimerized state and parallel TK transphosphorylation [26].

**Figure 1:**
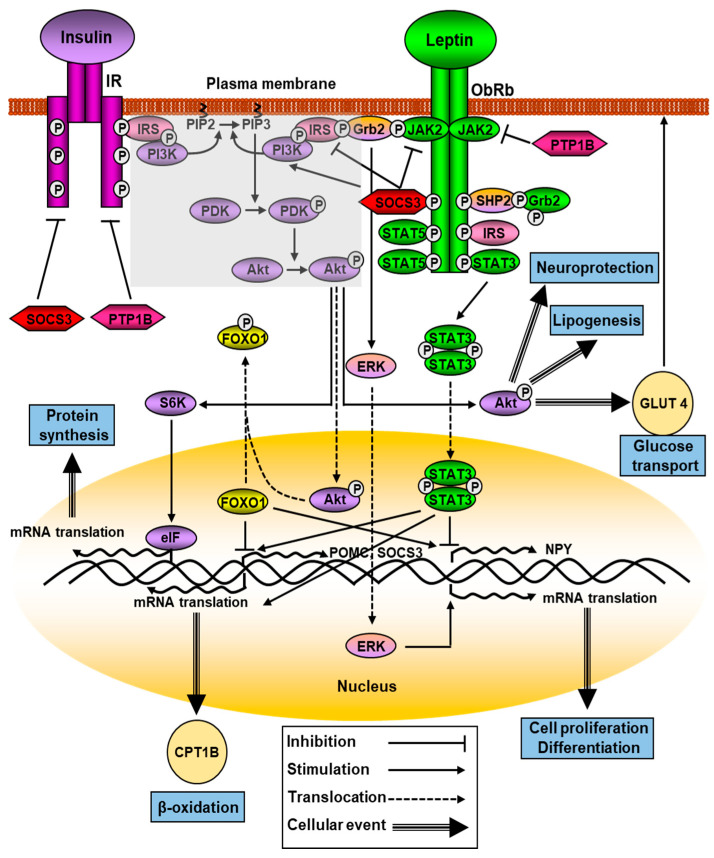
Multiple signaling pathways of leptin and its downstream effectors. ObRb oligomerization (for the clarity of the figure, only dimerization it is shown) activates JAK2/STAT3, PI3K, and ERK pathways. Activated JAK2 kinase induces Tyr residues of ObRb, that activates STAT-3 and -5. ObRb phosphorylation recruits SHP2 and Grb2 and increases ERK signaling. Crosstalk of leptin and insulin signaling is represented by the shaded area. See Section 2.1 for details.

The main intracellular signaling pathway for the ObRb is the JAK-STAT pathway. The conserved ObRb Y1138 motif serves as a binding site for the SH2 domain of STAT3. When phosphorylated by JAKs on Y705, STAT3 is translocated as dimers to the nucleus, where it regulates the expression of different STAT3-responsive target genes that mediate leptin’s primary actions, including suppressor of cytokine signaling (SOCS)3. This molecule acts as a potent negative regulator of the JAK/STAT signaling pathway, thereby forming a negative feedback loop (Figure 1). This inhibition mechanism through SOCS3 seems to be linked to Y985 of ObRb, since mutation of this residue prevents the feedback mechanism [27]. Increased SOC3 activation, which would in turn inhibit the JAK2-STAT3 signaling pathway activated by leptin receptors, is one of the main mechanisms involved in leptin resistance.

There are studies in mice where selective deletion of JAK2 in hepatocytes was shown to protect against steatosis and glucose intolerance induced by the intake of a high-fat diet [28], whereas patients with portal vein thrombosis associated with a point mutation in the JAK2 gene had a higher proportion of hepatocarcinoma [29]. Therefore, more studies are needed to clarify the role of changes in JAK2 signaling in pathophysiology, although numerous cases have been described that suggest that JAK2 mutations are related to carcinogenic processes [30].

Genetic inactivation of STAT3 causes hyperphagia and obesity, although it does not totally affect the regulation of energy homeostasis. Deletion of STAT in POMC neurons, which have a response element to this factor in their promoter, does not completely abolish leptin effects and causes only mild obesity. Thus, other cell types such as AgRP neurons are involved since their levels do not change in this situation [22]. Activating mutations in STAT3 have been related to different pathologies, including neutropenia [31] and processes of carcinogenesis and metastasis, where increased cell proliferation, migration, and survival stand out [32]. In contrast, dominant-negative mutations in this factor are related to a heterogeneous group of inborn errors of immunity that are associated with augmented infection susceptibility, eczema, and elevated serum IgE [33].

The ablation of SOCS3 in ObRb-expressing cells concomitantly reduces food intake, fat depots, and weight gain, increasing glucose tolerance and insulin sensitivity [34]. Moreover, deletion of SOCS3 in POMC neurons enhances glucose homeostasis and leptin sensitivity, whereas increased SOCS3 expression in these neurons results in decreased STAT3 signaling and leptin resistance. In addition, maternal obesity increased SOCS3 mRNA levels in the offspring, which was accompanied by reduced POMC expression, increased inflammation, and hypothalamic leptin resistance [35]. Few mutations in the SOCS3 gene have been described in humans. Several cases of gastric cancer have been reported, that seem to be more related to non-coding RNA-mediated gene silencing processes than to methylations affecting the functionality of SOCS3 [36]. In oncogenic diseases, such as glioblastoma multiforme, an increase in methylation of the SOCS3 gene has been detected, with a subsequent decrease in its expression [37], whereas overexpression of SOCS3 has been associated with changes in circulating miRNAs in children with obesity [38].

Phosphorylated Tyr985 operates as a docking site for the C-terminal SH2 domain of SHP2 (SH2-containing protein tyrosine phosphatase 2). Subsequently, SHP2 is phosphorylated by JAK and recruits growth factor receptor-bound protein 2, that precedes the activation of mitogen-activated protein kinase (MAPK) extracellular signal-regulated kinase 1/2 (ERK1/2) and the regulation of genes involved in proliferation and differentiation. The phosphatase activity of SHP2 is essential for ERK activation mediated by leptin [39]. Inhibition of ERK1/2 blocks the appetite-inhibiting actions of leptin, and deletion of SHP-2 in POMC neurons causes mild obesity, along with an imbalance of energy homeostasis [40]. Preclinical studies performed with allosteric SHP2 inhibitors were satisfactory, and combined treatments of SHP2 and ERK inhibitors resulted in enhanced antitumor benefits [41].

Leptin also activates STAT5 “in vitro” and “in vivo”. Activated STAT5 mainly binds to phosphorylated (p) Tyr1077, but also to a lesser extent to pTyr1138, of the ObRb [42]. However, mutations in these residues has only a slight effect on increasing food intake and body weight, suggesting that this intracellular mechanism plays a modest role in the control of metabolism by leptin. In fact, ablation of STAT5 in ObRb-expressing neurons failed to alter the energy balance [43], and STAT5 deficiency in mature adipocytes of obese mice was only associated with increased fat accumulation but was not related to improvement in glucose and lipid metabolism [44].

Leptin and insulin modulate metabolic processes by acting on both central and peripheral organs and share several signaling targets, with the activation of PI3K being the most important site of crosstalk between these hormones [45]. IRSs, mainly IRS-1 and-2, are enrolled by the ObRb via SH2B1, a cytoplasmatic protein that contains a SH2 domain, upregulating JAK2 kinase activity. SH2B1 mutant mice present hyperphagia, insulin resistance, and obesity, and hypothalamic overexpression of this protein increases leptin-signaling pathway activity that protects against high-fat-diet-induced obesity and metabolic syndromes [46]. After phosphorylation, IRSs bind to PI3K that is activated, phosphorylating Akt (Figure 1). Mutations in the regulatory (p85) and catalytic (p110) subunits of PI3K represent a driving force in the beginning and expansion of cancer [47].

Akt has a key role in numerous cellular processes, such as carbohydrate metabolism, and impairment of this pathway induces insulin resistance and can activate the synthesis of advanced glycation end products and reactive oxygen species, resulting in protein misfolding, mitochondrial dysfunction, and inflammation. It plays a critical role in diabetes and several neurodegenerative diseases [48]. Some Akt actions are mediated by Forkhead Box O1 (FoxO1) inhibition; thus, FoxO1 is unable to promote the transcription of NPY and to suppress POMC. This forkhead factor antagonizes STAT3 actions on these neuropeptides, and furthermore, it directly interacts with STAT3 to block leptin-induced POMC transcription [49]. Mechanistic target of rapamycin (mTOR), which is an energy sensor, is also activated by PI3K/Akt and promotes protein synthesis and cell proliferation when enough nutrients are available. Abnormal activation of this molecule due to changes in the levels of specific interleukins and growth factors is associated with the appearance of cancer [50]. Protein tyrosine phosphatase 1B (PTP1B), a negative regulator of the insulin signaling pathway, can also negatively regulate leptin signaling by dephosphorylating JAK2, whereas STAT3 phosphorylation was shown to be augmented in microglia in the arcuate nucleus of PTP1B knockout mice after a high-fat diet [51].

Leptin activates p70 S6 kinase (S6K) though mTOR. Selective inhibition of mTOR or S6K in the brain reduces the anorexigenic actions of leptin, whereas prolonged activation of S6K by insulin and nutrients inhibits PI3K-related signaling via negative feedback input to IRSs [52]. Leptin also induces the activity of phosphodiesterase 3B (PDE3B), resulting in a decrease in cAMP concentrations through the PI3K signaling pathway (Figure 1). Hypothalamic PDE3B deficiency modifies body weight and glucose homeostasis in mice, increasing insulin sensitivity, with these changes being independent of alterations in central NPY and POMC mRNA levels [53]. Leptin also augments glucose uptake in liver and muscle, and blocks acetyl-CoA carboxylase activity by activating AMP-activated protein kinase (AMPK), protecting non-adipose tissue against lipotoxicity, but inhibits AMPK activity in the hypothalamus, diminishing food intake and body weight. This activation requires the intervention of JAK2 and does not require the intracellular phosphotyrosine motifs in the ObRb.

Leptin possesses structural homology with the cytokines of the long-chain helical family that includes several proinflammatory interleukins, and ObRb has similarities with members of the class I cytokine receptor superfamily, which includes the receptor for IL-6, among other factors that can influence the actions of leptin [54]. In fact, leptin signaling pathways interact with the actions of diverse inflammatory molecules including for example, TNF-α, IL-1, IL-2 or IL-6, which can modulate the leptin response, especially in certain pathological situations where there is some degree of inflammation such as obesity, diabetes, or cancer. On the other hand, the administration of specific antagonists of the leptin receptor, decreasing the signaling of this adipokine, have been shown to reduce the differentiation of proinflammatory cells [55]. Recently, possible mechanisms of different factors that could increase leptin resistance and the degree of inflammation have been identified, antagonizing the actions of leptin. The effects of leptin on insulin secretion were shown to be antagonized by TNF-α through reducing the expression of ObRb, and therefore, favoring the development of resistance to leptin action and the appearance of alterations in glucose homeostasis [56]. However, this crosstalk between leptin signaling and inflammatory processes also occurs in physiological situations that allow regulation of energy homeostasis and body weight [57].

There are different strategies to reverse states of resistance to the action of leptin, especially in obesity. Among them, feeding restriction, physical exercise, or partial gastrectomy have been proposed, as they reduce weight [58]. In addition, in physiological situations such as pregnancy, there is hyperleptinemia and leptin resistance, altering the function of different hypothalamic nuclei. This situation is mediated by prolactin and placental lactogen, which increases SOCS3 levels and decreases leptin transport. The result is an increase in energy intake and adiposity, a situation that is reversed after parturition. These situations are known as leptin resensititation [59]. Nevertheless, the dogma that all inflammatory cytokines aggravate obesity and associated comorbidities is questioned by the relationship between leptin and IL-1. Both leptin resistance and the absence of the receptor that mediates most of the actions of this interleukin (IL-1R1) are associated with obesity. Furthermore, this receptor mediates leptin resensitisation after the administration of celastrol, a potent anti-obesity agent, with the physical interaction between ObRb and IL-1R1 being the molecular mechanism involved [60].

### 2.2. Leptin Expression and Secretion

In humans, the LEP gene encoding for leptin has been localized on the 7 alpha 31.3 chromosome and is comprised of three exons separated by two introns. The leptin promoter has several conserved regions and point mutations in the CCAAT enhancer binding protein-α (C/EBP-α), TATA motifs and a consensus specificity protein 1 (Sp1) site were shown to decrease promoter activity, whereas mutation in a fourth factor-binding site, lipid transfer protein 1 (LP1), not only reduced promoter activity but also abolished protein binding [61]. Moreover, promoter binding sites for glucocorticoid receptor (GR), CREB, an E-box element that may bind sterol regulatory element-binding protein 1 c (SREBP1c), and Fos like 2 (FOSL2), a member of the AP-1 transcription factor family, have been identified [62].

Directed mutagenesis studies have allowed the characterization of two enhancers at the transcription start site. Both interact with the promoter and at least one of them is necessary for the basal expression of leptin. In the last years, studies demonstrated that leptin expression in adipose tissue may be regulated by redundant cis elements and trans factors interacting with the proximal promoter together with a long noncoding RNA [63]. Although this RNA sequence does not interact directly with these enhancers but with the promoter, it is necessary for the generation of mRNA, as lack of this noncoding region significantly reduces leptin expression.

Single-nucleotide polymorphisms (SNPs) in this noncoding region have been identified in obese individuals with low circulating leptin levels, which supports its possible role in the control of leptin expression [64]. By performing a genome-wide association study (GWAS), these authors identified several genetic loci gene in intronic regions linked to peripheral leptin levels, with most of them having a relationship that was independent of adiposity.

Most studies analyzing the mechanisms that control the production and secretion of leptin have been carried out in adipocytes, the main source of this hormone. Basal pro-duction is related to energy availability, i.e., adipocyte size, and it is modified by nutritional status, essentially through changes in mRNA levels. Thus, fasting decreases peripheral leptin levels, while feeding or obesity increases leptin levels. Leptin expression shows circadian variations and is positively regulated by many factors, such as glucose, insulin, and glucocorticoids, whereas several proinflammatory cytokines and β3-adrenergic agonists reduce leptin gene expression [65].

Insulin is reported to increase leptin expression through the activation of several transcription factors, such as C/EBP-α, SREBP1, and Sp1 [66]. The mechanism involves activation of PI3K and mechanistic target of rapamycin (mTOR). In addition, early growth response 1 (Egr1), a zinc finger transcription factor of a gene family that primarily participates in differentiation, growth control, and cancer progression, is expressed in adipocytes and is rapidly induced by nutrients and insulin, through direct binding to leptin gene promoters and stimulating transcription [67]. Glucocorticoids augment leptin mRNA levels in mammals and the combination of insulin and glucocorticoids increase leptin expression synergistically in adipocyte cultures [68]. This result suggests that these hormones could be the main regulators of this adipokine in humans.

Pro-inflammatory cytokines, such as interleukin (IL)-1β, IL-6, and tumor necrosis factor (TNF)-α, can have long-term influences on circulating leptin levels at the transcriptional level. Short-term effects of several inflammatory cytokines include a sudden in-crease in peripheral leptin levels, probably at the expense of a preformed pool [69]. Several factors inhibit leptin expression, such as growth hormone, free fatty acids, and the receptor agonists’ peroxisome proliferator-activated receptor-γ (PPAR-γ).

Epigenetic modifications play a crucial role in the regulation of leptin expression, not only during preadipocyte differentiation, but also in the regulation of mRNA levels in mature adipocytes. These processes involve various mechanisms, including modification of the accessibility to the leptin promoter through methylation and demethylation mechanisms, changes in the functionality of chromatin by post-transcriptional modifications of histones by mechanisms of acetylation, and ubiquitination in basic amino acids and finally microRNAs (miRNAs) [70]. Changes in methylation and miRNAs have been described both in patients with obesity and experimental models of obesity, whereas histone modifications were essentially reported in experimental models of obesity.

Demethylation of specific CpG islands distributed within a 317-bp sequence of the proximal leptin promoter occurs during adipogenesis and is associated with the onset of leptin expression [71]. It has also been reported that under basal conditions, methylation of CpG islands in the leptin promoter is elevated in 3T3-L1 adipocytes. However, after pretreatment with a DNA methyltransferase inhibitor, the addition of insulin increased leptin mRNA levels, whereas dexamethasone suppressed it [72]. In this regard, changes in insulin levels/sensitivity in patients with obesity correlated with DNA methylation in the promoter of the leptin gene [73]. With respect to histone modifications, inhibitors of his-tone deacetylase 6 are effective leptin sensitizers and anti-obesity agents in diet-induced obese mice. These inhibitors reduce food intake and fat stores, and improve glucose homeostasis [74].

These aspects mentioned above will be dealt with later in the sections corresponding to pathology. Epigenetic mechanisms such as DNA methylation and miRNAs are associated with microenvironmental changes that can increase the inflammatory status and associated diseases, such as obesity, diabetes, or cancer, among others. Studies in murine adipose tissue showed that synthesis of leptin is mainly modulated by food intake, fasting, insulin, and adrenergic agonists. Short-term starvation decreases its synthesis, with no apparent changes in transcription [75]. Activation of the sympathetic nervous system inhibits the synthesis of this adipokine; β-adrenergic agonists decrease the rate of synthesis and secretion, without modifying the content of mRNA. In addition, these compounds are reported to block the stimulatory effect of insulin “in vitro”, although some synergistic effects of insulin and isoproterenol in tissue explants has been described [76]. Food intake and insulin are two of the main factors involved in the post-transcriptional regulation of leptin. In fact, both can modify the start of the translation process. Leptin mRNA has a short 5′-untranslated region (UTR) that can double the rate of translation without changes in the messenger. It also has a 3′-UTR region with various structural motifs that regulate the stability of the mRNA and the translational efficiency. Insulin unblocks translational repression through mechanisms involving the interaction of both UTRs. In addition, in the 3′ region, there are different miRNA binding sites that can modulate the synthesis of this hormone [77]. Leptin expression, as well as its metabolic pathways, are regulated by various miRNAs and vice versa. Upon “in vitro” leptin stimulation, there is an increase in the expression of miR-21, miR-96, miR-31, and miR-182, reported to have oncogenic actions which have been reported, such as oncogenic miRNAs; in addition, leptin reduces tumor suppressor miRNAs (miR-143, miR-26b, miR-27b, miR-489) [78].

There is abundant data in the literature on miRNAs involved in resistance to hormonal action, obesity, and related pathologies, such as some cancers and lipodystrophy [79], showing a close relationship with the regulation of energy homeostasis [80]. Some miRNAs play a role in obesity and adipogenesis, including let-7, miR-15a, miR-17-92, miR-21, miR-27, miR-30, miR-31, miR-103, miR-107, miR-125b, miR-130, miR-138, miR-143, miR-150, miR-200, miR204/211, miR-210, miR-221, miR-222, miR-326, miR-335, miR-355, miR-378,miR-448, and miR-519d, amongst others [81].

An increase in circulating levels of lipids, hexoses, and amino acids modifies the production of leptin. Fatty acids block its synthesis, with this effect being related to the inhibition of insulin signaling, while carbohydrates stimulate its release. Branched-chain amino acids increase leptin synthesis, and as in insulin, the mechanism involves the triggering of the PI3K/mTOR pathway, and especially mTOR complex 1 (mTORC1), the main event mediating the effects of insulin and different nutrients on the process of leptin translation [82]. There is a close relationship between the mTORC1 complex and AMPK-mediated signaling pathways, which allows coupling of leptin synthesis with energy homeostasis mechanisms [83].

The mechanisms that regulate leptin secretion are not fully understood. Leptin is not found together with other compounds in secretion vesicles and it is not colocalized within the Golgi apparatus, which seems to indicate that there are specific storage vesicles for this adipokine. The inhibition of vesicular trafficking with different compounds suggests that leptin secretion is regulated in a classical way, similar to that of other hormones [84], but calcium and zinc modulate leptin secretion from adipocytes in a way that is dis-similar from its role in the exocytosis of many other polypeptidic hormones [85]. Different hormones and stimuli regulate leptin secretion. Insulin increases it, through a mechanism that seems to involve activation of phosphodiesterase III [86]. Other hormones, such as oxytocin and orexins, as well as different interleukins, also stimulate leptin secretion in various cell types. The mechanisms seem to involve an increase in glycemia and insu-linemia in most cases [87,88]. The endocrine stress axis also appears to be implicated in these mechanisms, as epinephrine augments leptin secretion in fish and this could be related to adjust glucose mobilization and energy homeostasis in an adaptive stress response [89]. Norepinephrine, lipolytic hormones increasing intracellular cAMP levels such as thyrotropin-stimulating hormone and adrenocorticotropic hormone and specific inhibitors of phosphodiesterase III, reduce insulin-stimulated leptin secretion, whereas β2-adrenoceptor deficiency augments leptin with a concomitant decrease in sympathetic tone [90].

## 3. Functionality of Leptin and Its Involvement in Pathology

Mutations in the genes for leptin and its receptor, as well as in some of the associated signaling targets, are related to several pathological states. Likewise, changes in the transcription and translation processes can alter local and peripheral levels of this adipokine, modifying its functionality. Finally, changes in the levels of its receptors, especially ObRb, can also be modified in different diseases, most of them related to various degrees of inflammation. Consequently, its implication in diverse central and peripheral disorders is wide, given the plethora of actions of this cytokine.

### 3.1. Leptin-Associated Central Dysfunctions

The originally described central actions of leptin were mainly related to the regulation of food intake and energy homeostasis at the hypothalamic level [91]. Subsequently, it was reported that leptin is also involved in neurogenesis and has neuroprotective actions [92]. Thus, changes in its functionality may be related to alterations in the regulation of body weight, or different neurodegenerative processes.

#### 3.1.1. Central Leptin Resistance and Dysregulation of Energy Homeostasis

Numerous mechanisms by which leptin resistance is caused have been described, but they are not fully clarified. Among these, a decrease in the intracellular signaling pathway of leptin in different neurons of the central nervous system (CNS) stands out, especially in the hypothalamus. This may be due to changes in the hypothalamic levels of ObRb or also to mutations in its intracellular domain that decrease the triggering of signaling after leptin binding [93]. This response is also altered by the increase in feedback inhibitors. This may be associated with an effect of leptin itself in the increase of leptin resistance, with this state called “leptin-induced leptin resistance” [94]. A prolonged augmentation of leptin in the CNS promotes an increase in STAT3 phosphorylation and translocation to the nucleus, where it activates SOCS3 transcription.

A decrease in positive regulators of the leptin cascade attenuates intracellular signaling. For example, SH2B1, a cytoplasmic adaptor protein, binds via its SH2 domain to JAK2, stimulating this leptin-related pathway. Its deletion provokes metabolic disorders in SH2B1 KO mice, including leptin resistance, obesity, and glucose intolerance whereas restoration of SH2B1 corrected these metabolic disturbances and improved JAK2-mediated leptin signaling and regulation of hypothalamic orexigenic neuropeptides [95]. In addition, pathogenic variants in SH2B1 in young adults with severe obesity corroborate the role of this adaptor protein [96]. Autophagy seems to be involved in leptin resistance as hypothalamic ablation of autophagy-related protein 7 augmented food intake, generating leptin resistance and obesity. On the other hand, the specific deletion of this factor in AgRP neurons reduced food intake and weight, with a possible increase in sensitivity to the actions of leptin [97]. Therefore, it is suggested that an endoplasmic reticulum stress-autophagy pathway regulates hypothalamic development and energy balance in leptin-deficient mice [98].

Hypothalamic inflammation plays a key role in the generation of central insulin and leptin resistance with TNF-α and IL-6 involved in the maintenance of this situation. Insulin resistance also aggravates leptin resistance, given the crosstalking between these hormones, which is essential for maintenance of normal healthy energy homeostasis [99]. C1qTNF-related protein 4 (CTRP4) acts in the hypothalamus to modulate food intake and exerts anti-inflammatory effects on several cell types. Overexpression of CTRP4 in the hypothalamus of high-fat-diet-induced obese mice reduced central TNF-α and IL-6 levels, being consistent with the suppression of nuclear factor kappa-light-chain-enhancer of activated B cells (NFκB) signaling and restoring of leptin sensitivity [100].

Another key factor in the appearance of central resistance to leptin is impairment of its transport to the brain across the blood–brain barrier. This transport is dependent on the ObRb and is saturable, so if there are hyperleptinemia, the saturation of the receptor reduces its transport, and lower local levels into the brain may generate leptin resistance [101]. However, this point is controversial, since there are data that suggest an ObRb-independent transport mechanism and, in addition, astrocytes may also regulate leptin transport across the blood-brain barrier mediated by leptin receptors in a mechanism independent of reduction of paracellular permeability [102]. Hypertriglyceridemia specifically inhibits leptin transport across the blood–brain barrier [103]. Tanycytes, a specialized glial cell located in the medial eminence of the third ventricle of the hypothalamus, are involved in the transport of leptin and other hormones into the cerebrospinal fluid and play a role in the pathophysiology of central insulin resistance. These cells do not express the leptin receptor and thus, the mechanism by which this transport occurs is partially independent of the leptin receptor [104]. The recent characterization of new factors such as the cationic amino acid transporter-1, that increases leptin transport, suggests additional mechanisms for leptin transport. In this regard, the treatment of brain microvascular endothelial cell-like cells with substrates for this transporter generated a significant increase in leptin transport [105]. All of these factors mentioned above are involved in changes in the expression of the different neuropeptides that regulate energy intake and homeostasis, and in a prolonged situation, this causes metabolic disorders by disrupting the crosstalk between the hypothalamus and periphery, a situation that can be reversed with some antilipemic drugs that reduce body weight [106].

#### 3.1.2. Leptin, Neurogenesis, and Neuroprotection

Although leptin receptors were initially identified in hypothalamic neurons, they are widely distributed in different cell types of the cerebral cortex and hippocampus, areas involved in cognition and memory processes. Expression of ObRb in neural precursor cells is necessary for preserving an adequate body weight in addition to the differentiation of these precursors into neural or glial cells after birth [107]. Leptin is an activator of transcription through triggering CREB protein, that is required for leptin-stimulated synapse formation and also augments the expression of miRNA-132, a well-known CREB target, which is necessary for leptin-induced synaptogenesis [108]. CREB regulates the expression of numerous genes in neurons, including those that modulate synaptic development.

Leptin participates in the growth of hippocampal neurons and formation of dendritic spines in the hippocampal neurons, and this results in the enhancement of glutamatergic synaptogenesis during neonatal development [109]. This action is also mediated, at least in part, by the deubiquitinase ubiquitin-specific protease 8 that is accomplished with ObRb. Acute leptin stimulation increases this activity and mRNA levels through CREB-dependent transcription [110]. Surface expression of N-methyl D-aspartate (NMDA) receptor subtype 2B (NR2B) is also regulated by leptin, and after its activation forms a complex with ObRb [111]. NMDA is the major excitatory neurotransmitter in the mammalian CNS, and it is implicated in modulating functions such as learning, memory processing, pain perception, and feeding behaviors.

Genetically obese rodents (*db*/*db* mice, *fa*/*fa* rats) exhibit impairments in hippocampal memory processes showing deficits in spatial memory tasks detected in the Morris water maze [112]. Moreover, leptin infusion improves spatial learning and behavioral performance in mice and controls excitatory synaptic transmission in the hippocampus, increasing long-term potentiation and reducing long-term depression [113]. In this manner, this adipokine increases the efficiency of excitatory synaptic transmission, and upgrades learning and memory.

Leptin also has various neuroprotective actions and has been reported to reduce damage in models of glucose deprivation and transient cerebral ischemia. Experimental data connect metabolic deterioration and Parkinson’s disease (PD) with a dysregulation of central and peripheral neuroinflammatory networks mediated by several adipokines, particularly by leptin. Leptin had protective effects in an experimental model of PD, generated by a mitochondrial neurotoxin [114], and compounds that increase leptin signaling in a 6-hydroxydopamine rat model of PD attenuate neuronal apoptosis of substantia nigra [115].

Mitochondrial dysfunction has an established role in the development of Alzheimer’s disease (AD) pathophysiology. Leptin reduces depolarization of the mitochondrial membrane and mitochondrial fragmentation induced by amyloid β (Aβ). Leptin restrains up-regulation of the mitochondrial fission protein, Fis1, and down-regulation of the fusion protein, Mfn2, and increases the expression and activity of antioxidant enzymes [116]. Chronic leptin treatment of transgenic mice overexpressing amyloid precursor protein (APP) reduced brain concentrations of Aβ and phosphorylated tau, improving memory [117], and acute central leptin infusion increased brain somatostatin [118] with a concomitant uprising of CREB, involved in memory processes. Somatostatin is a neuropeptide involved in cognitive processes and is reduced in the hippocampus of experimental models of AD [119] and in AD patients [120]. Moreover, chronic central leptin treatment reduces Aβ-mediated impairment in memory and suppression of hippocampal LTP [121].

Leptin exerts neuroprotective effects and improves chronic sleep-deprivation-induced depressive-like behaviors. Animal models of post-traumatic stress disorders present activation of NLRP3 inflammasome in astrocytes sorted from glial fibrillary acidic protein (GFAP) transgenic mice, while administration of leptin markedly suppressed the activation of astrocytic NLRP3 inflammasome. Leptin effectively improved these behavioral alterations including cognitive impairments and depressive-like conducts. These actions of leptin were facilitated by STAT3 [122]. An increase in STAT3 also mediates the effect of leptin in promoting angiogenesis after hemorrhage. Leptin infusion promoted a dose-dependent effect on vascular endothelial cell survival and proliferation after intracerebral hemorrhage in rodents [8].

#### 3.1.3. Neurological Diseases

Clinical findings indicate that insulin and leptin resistance are related to cognitive deficits and neuropsychiatric disorders, and interestingly, these analyses propose that the associated deficits in neuroplasticity can be reversed by restoration of insulin and leptin sensitivity [123]. Parkinson’s disease, the second most common neurodegenerative disease, is characterized by gradual degeneration of dopaminergic (DA) neurons in the substantia nigra pars compacta. Leptin reversed the loss of dopaminergic neurons in an experimental model of PD that employed the dopamine cell-specific neurotoxin, 6-hydroxydopamine (6-OHDA). The neuroprotective effects occurred via ObR receptor activation and involved activation of JAK-STAT, ERK, and growth factor receptor-bound protein 2 (GRB2) [124]. Leptin augments brain-derived neurotrophic factor (BDNF) following leptin receptor activation, with this factor being a key factor for survival of dopaminergic neurons, which is also diminished in PD [125]. As leptin and BDNF activate shared signaling pathways, such as PI3K and MAPK/ERK and some intermediary molecules, including SH2 and GRB2, leptin could induce positive feedback by increasing BDNF expression. In a recent report [126], 10 PD-related hub genes, including JAK2, ObRb and IRS2, among others, are involved in AMPK and leptin signaling.

Mitochondrial dysfunction and oxidative stress are pathogenic processes involved in PD. Cells control such stress via several antioxidant factors. One family of proteins is the mitochondrial uncoupling proteins (UCPs), which are anion carriers located in the mitochondrial inner membrane. Leptin can preserve neuronal survival acting via UCP2 against mitochondrial membrane potential positive-induced toxicity, commonly used in experimental Parkinsonian models, by maintaining ATP levels and membrane potential [127]. These data indicate that leptin could have potential as a therapeutic target in PD, but research in this field is limited and generates controversies. In this regard, serum leptin levels in PD patients were non-significantly lower than controls in a meta-analysis study [128], although it was found that peripheral leptin levels in unintended weight loss PD patients were lower than those with stable weight [129].

There are data on the relationship between depression and leptin status. Animal studies of chronic stress report lower levels of leptin, and central infusion of this adipokine had antidepressant effects. Deletion of ObRb induces depression-like behaviors, suggesting that intracellular leptin signaling is implicated in the molecular mechanism of anti-depressant action of this cytokine [130]. Clinical trials examining the relationship between depressive processes and leptin levels are inconsistent. Some studies showed lower plasma levels of leptin in these patients; however, in cases of major depression disorder, serum concentrations were higher than in control subjects [131]. In another study, leptin was analyzed in depressive patients with therapy, finding a relationship between the combination of serum leptin and number of behavior disorders, suggesting that leptin levels can be a predictor of treatment responses in these patients [132].

There are data suggesting that leptin might play a role in AD. The decrease in plasma leptin levels with age has been associated with an increased risk of cognitive loss and the possible development of this disease [133]. In fact, this adipokine restrains tau phosphorylation, an essential premise for the formation of neurofibrillary tangles and studies in postmortem brains of AD patients showed a dysregulation of intracellular leptin signaling circuitry, with decreased ObRb mRNA levels in brain and colocalization of ObRb protein with neurofibrillary tangles [134], indicating a central resistance in this neurodegenerative disease.

An experimental model of AD in female rats treated chronically with Aβ showed a reduction in leptin levels and hippocampal leptin-related signaling, that was reverted with 17β-estradiol [135]. Indeed, 17β-estradiol administration is commonly related to better metabolic outcomes and neural benefits in old mice, with decreased β-amyloid burden, improved behavioral performance, and reduced microglial activation [136]. Estradiol is important for brain function; thus, 80% of postmenopausal women describe neurological symptoms including reduced cognitive performance. Preclinical evidence for neuroprotective effects of 17β-estradiol also indicate associations between menopause, cognitive aging, and AD, although there are discrepancies amongst clinical trials [137].

It has recently been reported that Aβ has a high affinity interaction with ObRb. Aβ binds allosterically to the extracellular domain of ObRb and significantly reduces different leptin signaling pathways, such as STAT3, PI3K, and ERK. In addition, the effect of leptin on POMC expression was reduced, indicating not only the disruption of the pathway, but also its effects on metabolism [138]. Chronic leptin treatment increases adult neurogenesis in transgenic mice that overexpress Aβ, with increased expression of ObRb in neurogenic niches of neural stem cells of these mice [139]. This adipokine reduced astrogliosis, microglial cell number, and the development of senile plaques, attenuating superoxide anion production and Aβ neurodegeneration.

Cytokines modulate neuronal plasticity and neurogenesis, intervening in cognitive processes. However, one must recall that leptin can promote inflammatory responses, in-creasing TNF and IL-6, which reduce synaptic plasticity and are related to the progression of AD and severity of cognitive impairment in these patients [140]. Leptin dysfunction in AD is hypothesized to be due to leptin resistance. Given that treating obesity with leptin is ineffective due to leptin resistance, it seems unlikely that AD will respond to leptin therapy as leptin resistance is thought to be an important factor in this disorder. Therefore, the use of leptin as a cognitive enhancer most likely has a reduced clinical application in these patients [141]. By itself, treatments that improve leptin sensitivity may be significantly more effective than solely replacing or supplementing this adipokine [142].

### 3.2. Leptin Status and Its Implication in Metabolic Pathologies

When leptin levels are in the normal range, this adipokine can exert its effects on energy intake and the numerous actions that regulate metabolism and contribute to maintaining energy homeostasis. Long-term changes in circulating leptin concentrations, either by excess or by deficiency, cause a series of disorders entangled in the pathogenesis of metabolic syndrome, diabetes mellitus, and cancer, among others.

#### 3.2.1. Obesity and Lipodystrophy

Obesity is usually accompanied by hyperleptinemia that is directly associated with an increase in body fat mass. This leads to leptin resistance, and the underlying mechanisms are diverse, including impaired ObRb signaling, changes in hypothalamic neural wiring, reduced brain leptin transport and ObRb trafficking, endoplasmic reticulum stress, and, frequently, low-grade inflammation [143]. Through genome-wide association studies and next-generation sequencing, approximately 500 obesity-related genes were identified, with mutations in some of these genes, including leptin and its receptor genes, POMC, MC4R, BDNF, prohormone convertase 1, unique homologue 1, and neurotrophic receptor tyrosine kinase type 2, having been reported to cause obesity [144]. In this section, the first two will be described.

About 5–8% of obese patients have a monogenic form of obesity. Complete leptin deficiency causes early-onset severe obesity. These patients have undetectable serum leptin levels and other endocrine abnormalities such as hypogonadotropic hypogonadism and hypothalamic hypothyroidism, endocrine findings that are due to leptin-hypothalamic linking intracellular signaling pathways implicated in the production of sex steroids and thyroid. Mechanistically, defects in the synthesis and/or secretion of leptin have been suggested and confirmed for several of these mutations. The administration of leptin to these patients causes a loss of fat and weight, as well as a signal of adequate satiety [145].

Mutations in the leptin receptor produce a monogenic form of obesity typified by hyperphagia and weight gain, with a prevalence of around 3% of children with obesity [146]. To date, different mutations of the human leptin receptor have been reported and, in most cases, subjects present severe obesity, alterations in immune function, and delayed puberty due to hypogonadotropic hypogonadism with serum leptin levels in the range predicted by their raised fat mass [147]. However, their clinical characteristics are reported to be less pronounced than patients presenting congenital leptin deficiency due to mutations in the leptin gene. Loss of leptin receptor function has been related to infertility, although a new recent mutation described in three generations demonstrated that all these patients were able to conceive [148].

Predisposition to the development of obesity in childhood and in adulthood can be affected by the milieu in the early stages of life. In animals, dietary changes during gestation produce alterations in the metabolism and body composition of the offspring, while in humans a high body mass index and an abnormal increase in gestational weight in-crease the risk of obesity in the descendants [149]. Adiposity may involve epigenetic regulation of different genes; indeed, in murine models, variations in the maternal diet vary the methylation of key metabolic genes, with the majority of these modifications persisting throughout life, thus modifying gene expression and the metabolic processes associated with these changes. For example, Lin et al. demonstrated that high-fat diet (HFD)-exposed offspring have hypomethylation in the leptin gene in subcutaneous and visceral adipose tissue [150]. In adult mice exposed long-term to a high-fat diet, DNA methylation of leptin and PPAR-γ promoters increased in gonadal adipose tissue; the same does not occur in subcutaneous adipose tissue [151]. In humans, changes in maternal dietary composition are associated with modifications in adiposity and bone mineral content with differential methylation of several promoters in the umbilical cord [152]. Moreover, in adolescents, differentially methylated CpG loci were associated with usual adiposity and most of them positively correlated with serum leptin [153], and leptin promoter methylation at birth and 12 months predicts weight and adiposity childhood [154].

Most studies find epigenetic variation associated with unfavorable exposures in utero, analyzing DNA methylation in genes, showing small methylation changed be-tween the compared conditions, less than 5% in case-control analysis. Both the proximal promoter of the leptin receptor and a part of its coding sequence are on a CpG island susceptible to epigenetic modifications during pregnancies with metabolic alterations. The a and b isoforms of the leptin receptor are expressed in the placenta of rodents and pregnant mice receiving diets rich in saturated fat showed a decrease in ObRa, suggesting a nutrient-availability regulatory mechanisms for the leptin receptor [155]. In humans, maternal obesity was shown to increase DNA methylation of the leptin promoter in the placenta, as well as to result in low levels of leptin receptor, although without differences in promoter DNA methylation in the fetus [156].

**Figure 2:**
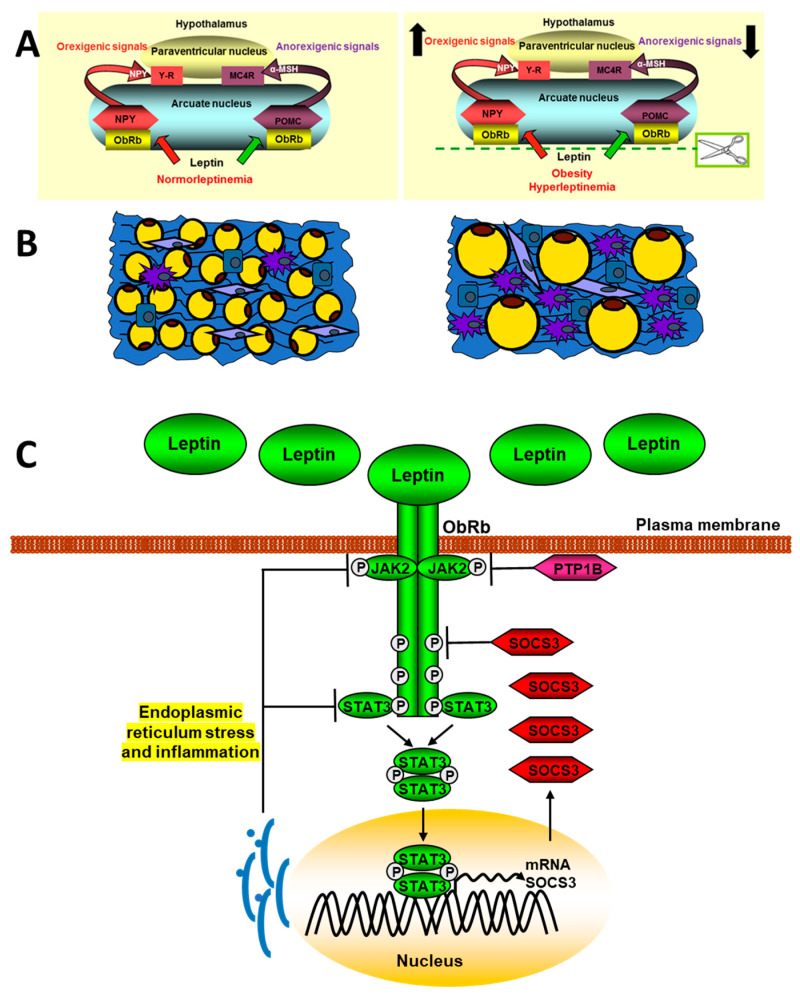
Schematic signaling pathways of leptin in obesity. The upper panel (**A**) represents a normal situation (**left**) and another with hyperleptinemia (**right**) and its effect on the expression of hypothalamic neuropeptides that regulate intake. The panel (**B**) shows adipose tissue in lean (**left**) or obese patients (**right**). The adipose tissue in obese subjets presents a hypertrophy of adipocytes, as well as a greater infiltration of macrophages and other inflammatory cells. In panel (**C**), the mechanism involved in the endoplasmic reticulum stress blocking of leptin signaling is indicated. See Section 2.1 and Section 3.2.1 for details.

The different types of SOCS are negative regulators of cytokine signaling through JAK/STAT. Thus, after the binding of leptin to its receptor and the activation of STAT3, SOCS3 is synthesized, which binds to the Tyr985 of the ObRb, preventing the phosphorylation of JAK2 [157]. Deletion of SOCS3 in neurons delay the onset of central leptin resistance in mice after high caloric diet intake [158]. Leptin and insulin share many signaling targets and one of them is PTP1B, a characteristic inhibitor of the insulin pathway. This phosphatase is contained to the cytoplasmic face of the endoplasmic reticulum and constrains leptin signaling by binding and subsequent dephosphorylation of JAK2. Mice with central PTP1B-deficiency are lean, sensitive to leptin, and partially protected from diet-induced obesity [159]. Changes in leptin signaling in obesity are shown in Figure 2.

Proinflammatory cytokines, as IL-20, are increased in obese patients and promotes an inflammatory environment by increasing other factors, such as TNF-α, monocyte chemotactic protein 1 (MCP-1), and leptin in adipocytes. Thus, this interleukin can increase insulin resistance by reducing glucose uptake through activation of the SOCS-3 pathway [160]. Excessive intake activates hypothalamic NFκB signaling through endoplasmic reticulum stress responses, connecting this stress to central inflammation [161]. IKK inhibition reduces leptin resistance by restoring JAK2-STAT3 and PI3K signaling in the hypothalamus of mice fed high-fat diets. Likewise, the central infusion of leptin shows a peripheral inhibition of the NFκB pathway together with the activation of PI3K [162].

The changes in the levels of ObRb in the cellular membrane depend on the synthesis, transport, internalization, and recycling, among other factors. These levels regulate the efficiency of leptin action and are known to increase after fasting and decrease after food intake, with leptin increasing hypothalamic expression of leptin receptor [163] but being unable to do so after the administration of diets rich in saturated fats. There are different molecules that regulate the exposure of ObRb in the cell membrane. Among these is endospanin-1, which is codified in humans by the same gene as the leptin receptor. This protein is increased by high-fat diets and regulates ObRb trafficking and cell surface exposure [164], whereas silencing of endospanin-1 in the arcuate nucleus of obese mice completely restores leptin responsiveness [165]. Thus, defective intracellular trafficking of the leptin receptor may cause leptin resistance seen in obese patients.

Leptin crosses the blood–brain barrier actively, being transport-saturable. In situations of obesity, the ratio of leptin in serum/cerebrospinal fluid decreases, showing impaired transport. ObRa isoform seems to be involved, but mutants in this isoform have minimal effect on leptin transport [166]. The median eminence is involved in leptin transport to the hypothalamus, with tanycytes mediating this action. Leptin administration activates ObRb in these cells, through activation of the ERK pathway and subsequent delivery to hypothalamic neurons. In murine models of obesity, leptin accumulates in the median eminence and experimental activation of ERK signaling restores its transport [167].

Resistance to the action of leptin can occur due to its excess, as well as a decrease in this adipokine. Lipodystrophy syndromes, which present a complete or partial absence of fat, are forms of leptin resistance due to hypoleptinemia. Molecular bases of these syndromes include, among others, modified differentiation of adipocytes, structure and regulation of the adipocyte lipid droplet, and early cellular senescence. These patients present metabolic complications such as insulin resistance, dyslipidemia, and fatty liver disease. A recent study in lipodystrophic mice treated with leptin showed a reduction in inflammation [168] and given the evidence of leptin deficiency in lipodystrophy syndromes; leptin replacement therapy has been contemplated as a treatment option. Long-term studies in children with lipodystrophy on the use of therapy with a methionylated human leptin analogue, metreleptin, have showed great improvements in the clinical situation and metabolic circumstances of these patients [169]. However, there are studies in obese patients with diabetes that established that metreleptin develops leptin tolerance; thus, this circumstance may limit the efficiency of this therapy [170].

MicroRNAs may be implicated in obesity and in modifications in leptin signaling activation. For example, miR-200a is overexpressed in the hypothalamus of *ob*/*ob* mice and its blockade restores both leptin and insulin signaling in the hypothalamus and liver [171]. In addition, leptin treatment significantly reduced hypothalamic miR-200a expression. Overexpression of pre-miR-200a causes a significant increase in miR-200a expression in SH-SY5Y human neuroblastoma cells, leading to considerable down-regulation of ObRb at both the mRNA and protein levels. In addition, miR-200a overexpression affects STAT-3 phosphorylation and Erk 1/2 phosphorylation in response to leptin. POMC expression is decreased in *ob*/*ob* mice, whereas miR-200a blockade in the hypothalamus increases its expression [171]. Moreover, Zhang et al. [172] found that leptin strongly up-regulates the expression of miR-21 in human mature adipocytes. It is known that miR-21 expression is upregulated by TNF-α, IL-6, leptin, resistin, and FFAs, but not by glucose. Leptin may affect obesity-associated insulin resistance by promoting miR-21 expression in adipocytes. Furthermore, miR-21 enhances the adipogenesis of human adipose tissue stromal cells by modulating TGF-β [173] and targeting STAT3 signaling [174]. These two signaling pathways interact with PPAR-γ signaling [175]. The miRNA let-7 is also related to obesity, with let-7 deficiency shown to prevent obesity induced by HFD feeding in mice, as well as hepatic steatosis accompanied by inhibition of the PPAR-α signaling [176]. A specific mouse line with hepatocyte-specific let-7b/c2 knockout shows that the direct target of let-7 ring finger protein 8 (Rnf8) is increased in let7b/c2ΔHep mouse liver and identified as a E3 ubiquitin ligase for obligate PPARα heterodimer partner retinoid X receptor α (RXRα). The study highlights a role for let-7 in the RNF8-RXRα regulatory axis, which regulates hepatic lipid catabolism [176].

#### 3.2.2. Diabetes

Serum adipokine levels exhibit similar bivariate relations with anthropometric variables in patients with type 1 diabetes mellitus (T1DM) to those in normal weight subjects. Circulating leptin is higher in overweight and obese patient with T1DM [177]. Leptin and soluble leptin receptor levels are increased in T1DM children and related to anthropometrics parameters, metabolic control, age of debut of diabetes, and kind of insulin therapy [178].

Animal studies show that leptin reduces hepatic glucose production by decreasing the MAPK phosphatase-3 (MKP-3) protein level via STAT3-enhanced MKP-3 and ERK combination [179], increases glucose uptake into tissues, modulates IGF-binding protein 2, and lowers blood glucose independent of hepatic leptin signaling [180]. This adipokine can also improve the diabetes of lipodystrophic mice independently of insulin [181], suggesting that leptin has therapeutic potential for the treatment of T1DM. PTP1B inhibits leptin signaling, and mice with T1DM induced by streptozotocin show hyperglycemia and reduced glucose metabolism, and leptin administration improves glucose metabolism in these mice. These effects were enhanced in mice with PTP1B deficiency [182]. Thus, inhibition of this leptin receptor inhibitor improves energy homeostasis in this metabolic disorder.

Central leptin action is sufficient to restore euglycemia in T1DM and is dependent on STAT3 activation, but not the release of fast-acting neurotransmitters such as glutamate and γ-aminobutyric acid [183]. In fact, central leptin can rescue T1DM hyperglycemia and recently, another mechanism has been proposed for this action. It was found that neurons expressing leptin receptor in the arcuate nucleus are selectively activated in this disease. These neurons have defective nutrient sensing and signs of energy deprivation that may be restored by leptin. The abnormal activation of these neurons due to energy deficiency as the neural basis for T1DM hyperglycemia and leptin action is mediated by inhibiting these neurons across withdrawing energy deprivation [184].

Hypoglycemia is the most serious complication of insulin therapy; therefore, adjunctive leptin treatment could reduce the severity of these episodes; indeed, the combination of insulin and leptin in T1DM mice was shown to improve glycemic stability. In addition, leptin may also have insulin-independent effects to reverse hyperglycemia and ketoacidosis in poorly controlled T1DM animal models. Leptin reduces hyperphagia and insulin resistance in liver and muscle in patients with lipodystrophy [185], and this fact together the data mentioned above increases the interest in possible treatment of T1DM patients with this cytokine, as these patients often present with weight loss and hyperphagia as a consequence of complete or partial insulin deficiency. However, differences between T1DM mice and humans may affect the efficacy of leptin treatment. T1DM mice are leptin deficient due to decreased fat mass resulting from uncontrolled diabetes unlike humans with T1DM that receive insulin therapy. In addition, leptin treatment in T1DM patients with normal leptin levels may have a different effect on blood glucose than treatment in T1DM mice deficient in this hormone [186]. On the other hand, leptin therapy might also minimize the weight gain that is associated with increased doses of insulin. Consequently, additional studies will be needed to evaluate these possibilities.

Diabetes mellitus may also result primarily from a state of insulin resistance (type 2 diabetes mellitus, T2DM). More than 80% of subjects with T2DM are overweight [187]. In fact, an underlying pathophysiological condition of obesity and T2DM is the reduced biological response to insulin in peripheral tissues such as the liver, adipose tissue, and skeletal muscle. These organs, together with the pancreas, regulate glycemia. Nevertheless, this is a simplified view, as the brain is a crucial insulin target and plays a key role in glucose homeostasis. Above all, the arcuate nucleus is of critical importance for sensing of adiposity signals, not only insulin, but also leptin and circulating nutrients [188]. There is a partial intersection between neuronal populations that regulate energy balance and glucose homeostasis; hence, obesity and T2DM may have similar origins that are related to dysfunctions in the central nervous system [189].

The genetically induced hyperphagia and obesity in leptin- and leptin receptor-knockout mice, as well as in Zucker obese rats, are associated with the development of insulin resistance, T2DM, and abnormal leptin signaling. Studies in a new model of mice, BTBR. Cg-Lepob/WiscJ ob/ob (*BTBR ob*/*ob*) mice with sustained hyperglycemia at less for 20 weeks, present anatomical alterations in the brain–blood barrier that provokes an ab-normal entry of leptin into the brain, with subsequent reduced central leptin signaling, indicating again the role of the brain in this metabolic disease [190].

The Zucker Diabetic Sprague–Dawley (ZDSD) rat is another model for experimental studies of T2DM, with a characteristic phenotype that is independent of leptin receptor signaling [191]. This rat does not carry the leptin receptor mutation (*fa*/*fa*) and develops a prediabetic state that progresses to overt diabetes with age, with common morbidities, such as cardiomyopathy, that are associated with an increased risk occurrence in prediabetic patients. Clinical deteriorations associated with human T2DM and observed in the ZDSD rat include delayed wound healing, nephropathy, and neuropathy [192]. Accordingly, ZDSD rats may be an advantageous model for understanding molecular mechanisms and discovering potential new treatments for T2DM.

Changes in leptin-signaling-related molecules play a role in T2DM patients. The analysis of the association of SNPs with T2DM demonstrated that SOCS3 and JAK2 genes may be associated with T2DM, whereas interaction between the SOCS3, JAK2, and STAT3 are related to metabolic syndrome features [193]. T2DM is associated with central and peripheral inflammation [194,195] and the JAK/STAT signaling pathway mediates the effects of multiple cytokines, with abnormal signaling closely related to diabetic complications. In this context, aberrant leptin signaling can be implicated in the pathogenesis of this disease. Increased activation of JAK2 and STAT3, accompanied by elevated levels of SOCS3 contribute to the development of vascular complications by mediating inflammation associated with vascular endothelial cells [196]. It is important to highlight the growing evidence that indicates that subjects with type 2 diabetes are at higher risk for some common tumors, including cancers of the prostate, colon, breast, endometrium, pancreas, and liver [197]. Increased insulin levels may trigger the cancer associations of several additional risk factors [198], comprising high waist circumference, visceral fat, body mass index, sedentary lifestyle, and an inadequate food intake [199], among others.

MicroRNAs may be involved in changes in leptin-signaling activation in diabetes. For example, studies in experimental models of diabetes showed that miR-125a-5p may be a modulator of glycolipid metabolism in T2DM, by restraining lipogenesis and gluconeogenesis in the liver and increasing glycogen synthesis by targeting STAT3 [200]. Furthermore, several adipokines increase leptin concentrations and reduce concentrations of adiponectin, influencing insulin sensitivity and its related diseases. This process modifies expression of miRNAs, including let-7 [201]. Let-7 functions with RNA-binding proteins Lin28a/b that when is overexpressed in mice, provoking an insulin-sensitized state able to resist high-fat-diet-induced diabetes [202]. Overexpression of let-7 causes insulin resistance and impaired glucose tolerance in mice by repressing some components of the insulin-PI3K-mTOR pathway, such as IR, IRS2, PI3K interacting protein 1, Akt2, tuberous sclerosis complex 1, and rapamycin-insensitive companion of mTOR. In addition, these authors found that some targets of let-7 presented SNPs that were related to changes in the control of fasting glucose and T2DM [202]. Moreover, miR-26b is downregulated by leptin [36] and this miRNA was found to be reduced in visceral adipose tissue (VAT) in insulin-resistant adipocytes, obese rodent models, and human obesity [203]. Insulin-stimulated glucose uptake is promoted by miR-26b, as well as the translocation of insulin-stimulated glucose transporter type 4 to the plasma membrane in mature human adipocytes. miR-26b inhibits phosphatase and tensin homolog deleted on chromosome 10 (PTEN), its target gene altering Akt activation in response to insulin, which increases insulin sensitivity via PI3K/Akt pathway [204].

Epigenetic mechanisms such as the methylation of DNA are reported to be involved in the prenatal development of leptin and insulin resistance and these mechanisms may explain the risk of T2DM for subsequent generations. On this way, South Asians present an abnormal burden of type 2 diabetes that may be attributed to a “thin-fat-phenotype”. Asian ethnicity and gestational diabetes were associated with higher placental leptin gene methylation, with maternal glucose and lipid metabolism associated with placental methylation of the gene for this cytokine [205].

Different strategies have been used in the management of T2DM, including metformin and related drugs. Aerobic exercise is suggested as an effective non-pharmacological strategy to reduce cognitive disorders that have an increased risk in this disease [206]. In experimental models of T2DM, exercise results in an improvement in the cognitive disorders and this is mediated by an in increase in the AMPK/sirtuin 1 pathway, while AMPK inhibitors suppress the beneficial effect of this exercise [207]. Different phosphatases exert feedback inhibition on insulin signaling. Among these, PTP1B stands out, which also exerts an inhibition on leptin signaling. Drugs targeting PTP1B have given promising results in T2DM animal models [208], but due to limited information on their safety, it has not been possible to use them in patients. There is a close relationship between obesity and leptin resistance, which may precede or co-occur with T2DM. Therefore, leptin sensitivity may be a possible therapeutic target in this disease. Insulin sensitivity modulators in combination with amylin and/or GLP-1 analogues may be of interest due to their central activity in the hypothalamus [209].

#### 3.2.3. Cancer

More than twenty years ago, it was reported that both leptin mRNA and protein levels were increased in several breast cancer cell lines and breast tumors [210]. Afterwards, other studies showed changes in circulating leptin levels, together with a decrease in one or more isoforms of its receptor in different cancer types [211]. Subsequent findings established that leptin signaling was implicated in promotion of endometrial cancer cell proliferation by activating STAT3 and ERK2 pathways and that leptin-induced phosphorylation of ERK2 and Akt was determined by STAT3 activation [212].

Evidence links obesity to cancer, which may contribute to the increased morbidity and mortality associated with obesity. An excess of food intake causes an increase in fat depots, altering the balance between pro-inflammatory and anti-inflammatory cytokines and thus, increased levels of some proinflammatory cytokines associated with predicted cancer development [213]. Changes in macrophage composition can be an additional mechanism linking obesity with the initiation and progression of cancer. Proinflammatory macrophages have often been associated with a classically activated phenotype, which enhances the immune response and opposes tumorigenesis whereas anti-inflammatory macrophages alternatively adopt a phenotype that lessens immunity and promotes tumorigenesis. Leptin in obese patients may generate an alternative phenotype (M2) [214], but obesity is also related to a proinflammatory “metabolically activated” phenotype that is both mechanistically and functionally distinct from the classic (M1) phenotype. This metabolically activated phenotype is induced by saturated fatty acids released by insulin-resistant fat cells in obesity that are not only in fat but also in mammary fat and in other tissues, and secrete proinflammatory cytokines involved in tumorigenesis [215].

**Figure 3:**
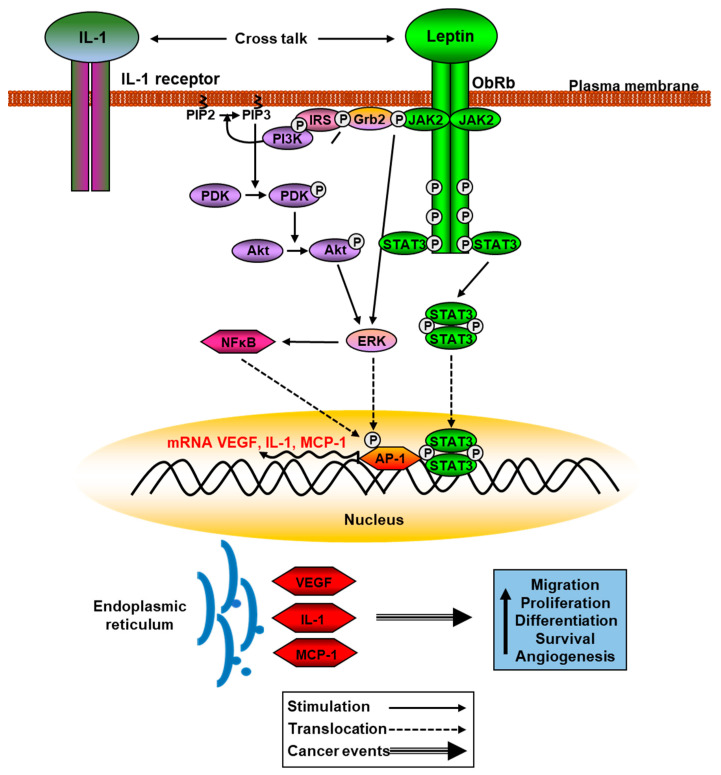
Schematic leptin signaling pathways involved in cancer. Phosphorylation of JAK2 and Akt activate ERK pathway that phosphorylates AP-1, increasing translation of VEGF, IL-1, and MCP-1, among other proinflammatory cytokines. See Section 2.1 and Section 3.2.3 for details.

Leptin and different isoforms of its receptor are abnormally expressed in cancer cells and tumor-adjacent areas. Methylation of the leptin receptor gene is lower in tumor samples than in adjacent non-tumor samples and its mRNA levels are directly related to the appearance of metastatic cells. The over-activation of ObRb leads to an increase in signaling in the JAK2/STAT3, PI3K, and ERKs pathways, which modulate the expression of genes related to cancer such as vascular endothelial growth factor (VEGF), cyclin D1, and cyclooxygenase-2 that favor angiogenesis, cell proliferation, and migration processes [216]. Leptin can also promote changes in the inflammatory environment by increasing the expression and secretion of different cytokines, which leads to increased migration and invasion of cancer cells in different organs [217]. Leptin may have synergistic actions with other cytokines in different types of cancers. Thus, these actions activate NFkB, increasing the synthesis of VEGF and MCP-1 by cancer cells leading to recruitment and activation of infiltrating tumor-associated macrophages, as well as the synthesis of other cytokines related to inflammation processes [218] (Figure 3).

Leptin actions in cancer progression are intensified through crosstalk with multiple oncogenes and proinflammatory interleukins adjacent to the tumor, favoring T-helper 1 responses. A relationship between cancer and obesity is clear and adipose chronic inflammation promotes cancer growth and epithelial–mesenchymal transition, related to morphological changes by decreasing contact between adjacent cells and promoting elongated morphological shape. Epithelial–mesenchymal transition is the ability of epithelial cells to change from a polarized morphology to a loose mesenchymal phenotype that accelerates cancer metastases [219]. The mechanism of this transition is the reduction of epithelial phenotype marker expression levels and the rise in the synthesis of vimentin. These changes lead to augmented cell mobility, tumor invasion, and metastasis.

This cytokine also plays a relevant role in the processes of invasion, metastasis, and angiogenesis. The molecular mechanisms involved are known in several types of tumors, but they have been more studied in breast cancer. Indeed, this adipokine promotes the migration and invasion of breast cancer cells through different mechanisms, one of them is the over-activation of enzymes implicated in the production of cholesterol esters [220]. Glucose is the source of energy in normal cells, but tumor cells can suffer a metabolic switch to de novo synthesis of different lipids, known as Warburg effect [221].

In fact, leptin is considered as a pro-tumorigenic protein known to activate PI3K, JAK/STAT3, and MAPK signaling pathways and also can up-regulate the process of autophagy in breast cancer, increasing the expression of several autophagy-related genes contributing to the suppression of apoptosis and growth of breast cancer cells [222]. These processes mediated by this adipokine involve the suppression of Bax and the activation of p53/FOXO signaling in different cancer cells. Leptin is implicated in the process of angiogenesis through activation of JAK2/STAT3, PI3K-Akt, and MAPK signaling pathways. The activation of these pathways promotes the synthesis of several angiogenic molecules, as VEGF [223]; the development of blood vessels; and the differentiation of endothelial cells, these events promoted by leptin closely linked to IL-1 signaling [224].

Genetic variants of the leptin and leptin receptor genes in cancer patients have been described in some studies and are associated with decreased risk of cancer susceptibility, smaller tumor size, less node and distant metastasis, and less distant metastasis [225], although other polymorphisms are related to an increase in tumorigenesis [226]. The leptin signaling cascade can be involved in changes in susceptibility to cancer processes and it has been suggested that genetic variations in JAK2 are involved in the generation of several signs of myeloproliferative neoplasms [227], as well as SOCS3 polymorphisms, that may favor the progression towards liver fibrosis and hepatocellular carcinoma [228]. Hypermethylation of SOCS3 has been reported to contribute to hepatocellular carcinoma development and progression [229].

Although it is a paradox, obesity can play a favorable role in tumorigenesis as a negative association between obesity and different neoplasms such as lung cancer, renal cell carcinoma, and Hodgkin’s lymphoma, among others, has been described, suggesting the protective role of obesity [230]. The first inhibitory effect of leptin treatment in human cancer cell lines was reported in pancreatic cells. Leptin levels were also correlated with a better prognosis in patients with colorectal cancer and leptin was reported to inhibit hepatocellular carcinoma proliferation in vitro through the p38-MAPK-dependent signaling pathway [231].

Obesity is related to an increase in leptin levels and a decrease in adiponectin concentrations, which alters miRNAs expressions, including let-7, miR-27, and miR-143, that are involved in both obesity [201] and cancer [232]. Furthermore, these miRNAs regulate PPARγ, which is known as a negative regulator of carcinogenesis. miR-31 inhibits adipogenesis by directly targeting C/EBPα [233] and represses cell proliferation and metastasis in breast cancer. Let-7 negatively regulates Harvey-RAS (H-Ras) and high-mobility group AT-hook 2 (HMGA2), suppressing differentiation and self-renewal of breast cancer stem-like cells [234]. Plasma leptin levels are significantly associated with miR-143 in obese adolescents [235]. This miRNA inhibits ERK5 (extracellular regulating kinase 5) [236], an ERK belonging to the MAPK family, and promotes cell differentiation [237], and ERK5 has a role in breast cancer prognosis [238]. Zhou et al. [239] performed luciferase assays and observed that miR-27 directly targeted the 3′ untranslated region (3′-UTR) of leptin. Leptin negatively regulates the expression of miR-27b through the PI3K and Akt cascade [240]. This miRNA is not expressed in breast cancer tissues and cell lines but is involved in anticancer drug sensitization in breast cancer cells in vitro and in vivo. Furthermore, miR-27 enhances the response to paclitaxel, an anticancer drug, by directly targeting Cbl proto-oncogene B and GRB2 genes, to inactivate PI3K/Akt and MAPK/ERK signaling pathways. It is suggested that miR-27b could be a potential biomarker in chemoresistance, clinicopathological features, and prognosis of breast cancer patients [241]. In addition, miR-21 plays a role in different pathways related to the promotion of cancer; this miRNA suppresses PTEN in PI3K/Akt pathway, programmed cell death protein 4, and NF-kB. On the other hand, curcumin decreases miR-21 levels [242], while treatment of breast cancer cells with oxidized LDL, which mimics a hyperlipidemic state, induces miR-21-mediated inflammation signaling and proliferation [243].

#### 3.2.4. Non-Alcoholic Fatty Liver and Steatohepatitis Diseases

Non-alcoholic fatty liver disease (NAFLD) represents a histological spectrum of liver disease that extends from isolated steatosis to steatohepatitis and cirrhosis and is the most frequent pathology amongst metabolic disorders and is related to obesity, diabetes, sedentary lifestyle, and Western diet. An excess of fatty deposits derives from this disease to non-alcoholic steatohepatitis (NASH), fibrosis, and is a frequent cause in the development of hepatocellular carcinoma. This pathology has a prevalence of 30% in Western countries and is defined as an accumulation of more than 5% of fat in hepatocytes. These triglycerides induce oxidative stress, alterations in metabolism, and mitochondrial damage that favor a proinflammatory environment [244], creating conditions for the development of the above-mentioned pathologies.

The activation of pattern recognition receptors, considered to be pivotal members of innate immunity, form a bridging pathway between innate and adaptive immune responses in hepatocytes, Kupffer cells, the resident macrophages of the liver, and other immune cells resulting in the production of proinflammatory cytokines and chemokines in NAFLD. Hyperactivation of the innate immune system through hepatic toll-like receptors (TLRs), members of pattern recognition receptors, stimulate the development and evolution of NAFLD by induction of proinflammatory and profibrogenic cytokines [245]. The production of these cytokines, such as TNF-α, various inflammatory interleukins, and MCP-1, among others, is mediated through activation of NFκB and MAPK [246].

Insulin resistance and increased leptin levels are early events in the development of NAFLD and NASH. Different studies and meta-analyses have shown that circulating leptin levels are associated with the severity of this metabolic disorder [247,248]. Leptin is considered an anti-steatosis hormone, since it reduces the accumulation of lipids, due to an increase in their catabolism, although lipid oxidation and oxidative phosphorylation can generate oxidative stress [249]. Nonetheless, leptin has effects on liver fibrosis in animal models [250] and ObRb is localized in Kupffer and stellate cells, indicating a delicate situation between lipid mobilization and possible development of fibrosis. Additionally, in situations of resistance to this cytokine, such as obesity, its action on the lipid metabolism is prevented, which promotes the development of NAFLD. In this regard, situations of leptin deficiency, such as lipodystrophy, are related to lipid accumulation in ectopic organs, such as the liver [251].

The experimental models for the study of NAFLD and NASH include those with deficiency in leptin or its receptor. Those deficient in leptin develop steatosis more frequently, but do not spontaneously develop fibrosis since leptin is a mediator for its development [252]. An interesting study showed the progression of hepatic lipid deposition and NASH in deficient-leptin gene rats [253]. Initially, simple steatosis in Lep^ΔI14/ΔI14^ Sprague–Dawley rats was observed with increased expression of the genes encoding for rate-limiting enzymes in lipid metabolism such as acetyl-CoA carboxylase and fatty acid synthase. Subsequently, with NASH progression, the authors found changes in the expression of genes involved in insulin resistance, inflammation, reactive oxygen species, and endoplasmic reticulum stress. As NASH phenotypes progress with age, higher activation of c-Jun N-terminal kinase and NFκB pathways and raised expression of cytokines and chemokines such as TNF- ɑ, IL-6, and IL-1β was reported. Another study showed the implication of STAT3 in liver diseases. A mutant variant of the patatin-like phospholipase domain-containing 3 drives development of steatosis and NASH by increasing triglycerides, reduction in n3 polyunsaturated fatty acids, and augmented ceramides with subsequent STAT3 phosphorylation and downstream inflammatory pathway activation [254].

High-fat diet provokes increased hepatic lipogenesis and inflammation, reduced fatty acid oxidation, and inflammation, with higher hepatic leptin levels and steatosis in mice. Reduction in leptin levels and steatosis are associated with weight loss mediated by the treatment of drugs that alleviate oxidative stress and reduce lipotoxicity [255]. Studies “in vitro” showed that activation of PPAR-γ and SOCS3 in Kupffer cells treated with saturated fatty acids participated in inflammation by releasing TNF-α and IL-6, and thus, this Kupffer cell dysfunction accelerated hepatocyte steatosis [256].

Changes in leptin signaling in patients with NAFLD are related to the pathophysiology of this disease; thus, the association of polymorphisms in the leptin receptor gene with NAFLD has been described [257]. In addition, mesenchymal stem cell-derived adipocytes from obese patients at different stages of NAFLD have impaired adipogenesis as liver steatosis augmented, showing a differential expression pattern in SOCS3, comparable to steatosis degree [258]. Different data in animals and humans show a relationship of certain miRNAs with hepatic metabolic homeostasis and leptin signaling. Many hepatic miRNAs located in the miR-379/miR-544 cluster were augmented in leptin-receptor-deficient type 2 mice and genetic mutation of this cluster in mice showed resistance to high-fat-diet-induced obesity with lower hypertriglyceridemia and steatosis [259]. Several compounds in modern diets are risk factors for the development of NAFLD and hepatocellular carcinoma, where various miRNAs are involved, with miR-650 being the most potent for the development of these diseases. SOCS3 is directly regulated by miR-650 and its suppression regulates the activation of the JAK2/STAT3 signaling pathway [260].

### 3.3. Other Diseases

The widespread peripheral expression of leptin and its receptor may account for additional anorectic and obesogenic effects and the peripheral pleiotropic effects of leptin could explain, at least in part, the comorbidities related to increased fat depots in numerous organs. In fact, leptin is implicated in the pathophysiology of bone and muscle, among many other organs. Leptin is considered as an inhibitor of bone formation, with bone loss being greater in subjects with a high body mass index, and particularly in people resistant to insulin or with T2DM. Different studies show that intracerebroventricular (ICV) administration of leptin reduces the volume of trabecular bone, although it is not known whether this is due to a direct action of leptin or through the sympathetic nervous system (SNS) [261]. The hypothesis of these central leptin actions through this system seems to be reasonable, since the signaling of leptin in serotonergic neurons of ventromedial hypothalamus recruits two mediators to inhibit bone mass accrual, CART and the SNS [262], also supported by the clinical observation that patients with high sympathetic activity develop severe osteoporosis. In addition, lipodystrophic mice deficient in leptin or leptin receptor show an increase in bone mass [263] and transplantation of fat from control animals decreases bone density, while adipose tissue from leptin or leptin receptor knockouts do not present changes in this parameter.

There are conflicting data, since the binding of leptin to its receptors on osteoclasts and osteoblasts induces the synthesis of the bone matrix, promoting the differentiation of osteoblasts and the synthesis of type I collagen [264]. Likewise, this adipokine improves the collagenolytic properties in cartilage explant cultures through the activation of matrix metalloproteinases and activation of STAT3 transcription and also inhibited osteoclast formation by increasing osteoprotegerin concentrations. These apparently discordant data may be due to their interaction with other factors, since leptin acts together with other hypothalamic factors, IGF-1, and estrogens [265], whereas a reduction in leptin levels is related to lower bone mineral density [266].

Administration of leptin to leptin-deficient *ob*/*ob* mice augments bone mineral density and content, as well as marrow adipocyte number and mineral apposition rate [267]. Osteoblast/osteocyte-selective STAT3 KO mice have lower bone mineral density and a reduced relative bone formation rate with an increased level of reactive oxygen species in osteoblasts [268], and patients with loss of function mutations in STAT3 have a variety of skeletal manifestations, including scoliosis, osteoporosis, and minimal trauma fractures [269]. In summary, the mechanisms by which leptin modulates the acquisition and reabsorption of bone mass and the interaction between the regulation of energy homeostasis and bone physiology are partially understood and require further research.

Leptin seems to have some beneficial effects on the heart, since the elimination of ObRb worsens cardiac function [270] and studies in cardiomyocytes indicate that this cytokine increases the capture and oxidation of fatty acids, mediated by the activation of AMPK and STAT3. However, leptin increases the proliferation of vascular smooth muscle cells and increases blood pressure, and its dysregulation also induces cardiac hypertrophy through the activation of JAK2/STAT3 and MAPK signaling pathways [271].

In skeletal muscle, leptin triggers AMPK and increases fatty acid uptake and oxidation and lessens triglyceride synthesis [272]. Central leptin infusion increases skeletal muscle size, proliferation and fiber area. ICV infusion also augments carnitine palmitoyltransferase-1b expression and mitochondrial respiratory chain complexes and reduces non-esterified fatty acids through activation of insulin signaling [273]. Leptin-deficient *ob*/*ob* mice have an increase in enzymes involved in lipid anabolism in skeletal muscle, providing evidence against reduced fatty acid oxidation in lipid-induced insulin resistance and showing a phenotype of “slow fiber type” [274]. The existence of polymorphisms in the leptin receptor gene has been associated with intramuscular fat content in mammals [275].

Sarcopenia is a common muscular affection among ageing people and recently, it has been identified as the skeletal muscle expression of the manifestations of metabolic syndrome. The prevalence of sarcopenia is increasing in parallel with visceral obesity, to which it is tightly associated in cancer patients [276]. Higher plasma leptin levels appear to be directly related to greater muscle mass and function, and conversely, in physically frail individuals with low body mass; the presence of decreased levels of leptin, associated with weight loss, could reveal a state of energy deprivation related to physical fragility [277]. Aging alters the expression of more than 50 miRNAs in muscle, some of them explicitly associated with age-related muscle atrophy. Treatment of aged mice with leptin augments muscle mass and fiber size in these animals. Likewise, the expression of 37 miRNAs was changed in the muscle of these treated mice [278]. Particularly, leptin enhances the expression of miR-31 and miR-223, both described to be elevated during processes of muscle regeneration and repair.

## 4. Closing Remarks

Circulating leptin levels can reflect the effectiveness of leptin, and consequently, leptin-deficient mice present marked responses to exogenous leptin, whereas hyperleptinemia causes lessened leptin response. The main function of leptin is to regulate the balance between food intake and energy expenditure, serving as a marker of long-term energy stores. When fat depots decrease, leptin production and its crossing of the blood–brain barrier diminishes. The CNS increases hunger while also promoting endocrine and autonomic machinery, and growth. When food intake increases and adipose tissue accumulation is excessive, there is an increase in the synthesis and secretion of leptin into the bloodstream. This signal reduces food intake and an increase in energy expenditure to counteract the excess of energy, as long as leptin insensitivity has not developed.

Leptin is a pleiotropic cytokine that exerts multiple actions, most of them related to the modulation of energy homeostasis. Leptin receptors have been described in many tis-sues and organs, and after leptin binding, multiple signaling pathways can be activated and interact with those of other hormones and cytokines. Each of these pathways can control specific aspects of leptin action and physiology, which explains the plethora of actions exerted by this adipokine.

The mechanisms that regulate the synthesis and secretion of leptin are finely con-trolled, allowing a physiological adjustment with other hormones and factors that synchronize the efficiency of leptin by modulating the mechanisms of transcription, translation, posttranscriptional modifications, and finally, the mechanisms of leptin release. Dysregulation of these processes, as well as subsequent events where other factors interact with this adipokine, are implicated in many of the diseases discussed in this review. The increase in leptin levels caused by overfeeding provides a physiological signal that causes leptin resistance in obesity, as well as in other central and peripheral pathologies. The mechanism of regulation of energy homeostasis are partially affected as leptin does not adequately cross the blood–brain barrier, and in addition, peripheral leptin actions also fail due to the increase in its signaling feedback effectors. Although this dogma of leptin resistance is true in many situations, this aspect of leptin physiology is not fully characterized.

In this way, as mentioned above, many other signals, such as insulin and other hormones, contribute to the control of food intake and to the pleiotropic actions of leptin. For this reason, the crosstalk that occurs in the signaling and the partial redundancy between these factors also make difficult the contribution of leptin actions in states of leptin resistance and pathophysiological situations analyzed in this review.

Recombinant leptin is under investigation for the treatment of both hypoleptinemia and hyperleptinemia-related syndromes. However, leptin replacement has only been shown to reverse obesity in leptin-deficient conditions and its use in obese individuals with elevated leptin levels shows null or limited efficacy. Efforts are focused on the search for leptin-sensitizing agents that may activate its different signaling pathways, and therefore, could augment the effects of this adipokine.

In summary, leptin has emerged in recent decades as a crucial hormone secreted mainly by adipose tissue that regulates food intake and energy homeostasis. The subsequent discovery of receptors for this adipokine, as well as its synthesis in numerous tis-sues, has allowed us to analyze its metabolic effects, as well as other functions, such as cell growth regulation and neuroprotective functions, among others. Obesity is a global problem and its relationship with cancer is complex and multifactorial. The current challenge nowadays is restoring leptin sensitivity by the future application of new-generation leptin analogs and enhancing leptin-related thermogenic responses in obesity. In addition, this adipokine is a key element in the inflammatory response to certain diets. Thus, the study of the effect of food components on inflammation and resistance to leptin action may provide the basis for the potential use of nutraceuticals. In cancer diseases, it is necessary to know in detail the cellular events promoted by leptin, and thus act against these targets that promote invasion, migration, metastasis, and angiogenic processes. Future directions to improve our understanding of leptin dysregulation and associated clinical diseases are needed to establish the potential of leptin as a diagnostic and prognostic biomarker and to find possible therapeutic targets against neurological and metabolic pathologies.

## Data Availability

Not applicable.
